# Aggressive treatment may be needed for idiopathic membranous nephropathy with focal segmental glomerulosclerosis lesions

**DOI:** 10.1590/1806-9282.20230871

**Published:** 2024-05-03

**Authors:** Ping Cheng, Qionghong Xie, Shaojun Liu, Xiaobin Liu, Liang Wang, Chuan-Ming Hao

**Affiliations:** 1 Huashan Hospital Affiliated to Fudan University, Department of Nephrology – Shanghai, China.; 2 Wuxi People's Hospital Affiliated to Nanjing Medical University, Department of Nephrology – Jiangsu, China.

**Keywords:** Idiopathic membranous nephropathy (IMN), Focal segmental glomerulosclerosis (FSGS), Treatment, Phospholipase A_2_ receptor (PLA_2_R)

## Abstract

**OBJECTIVE:**

The purpose of this study was to analyze the clinical, pathological, prognostic features and treatment response of the coexistence of focal segmental glomerulosclerosis lesions with idiopathic membranous nephropathy.

**METHODS:**

This is a two-center retrospective cohort study. Patients of idiopathic membranous nephropathy were enrolled and divided into two groups with or without focal segmental glomerulosclerosis lesions according to the renal biopsy. Laboratory data and pathological manifestation were compared. Renal phospholipase A_2_ receptor was detected by immunofluorescence. During the follow-up, the effects of different therapies and renal function were estimated.

**RESULTS:**

A total of 236 patients were finally enrolled in this study, of which 60 and 176 idiopathic membranous nephropathy patients were enrolled in the FSGS+ and FSGS- groups, respectively. The FSGS+ group showed a higher percentage of hypertension history (38.3 vs. 20.0%, p=0.004), with a significantly higher level of systolic pressure [137 (120, 160) mmHg vs. 130 (120, 140) mmHg, p=0.009]. Main laboratory findings, including serial albumin (20.4±7.8 g/L vs. 24.5±6.7 g/L, p<0.001), 24-h proteinuria [5.61 (3.10, 7.87) g/day vs. 3.82 (2.31, 5.79) g/day, p=0.002], serial creatinine [80.8 (65.8, 97.9) μmol/L vs. 72.0 (58.7, 84.9) μmol/L, p=0.003], and estimated glomerular filtration rate [86 (66, 101) mL/min/1.73 m^2^ vs. 95 (81, 108) mL/min/1.73 m^2^, p=0.007] showed significant differences between the two groups. Pathologically, patients with focal segmental glomerulosclerosis lesions appeared with a higher percentage of crescents, a more severe degree of interstitial fibrosis, and a higher level of membranous nephropathy stage. Renal phospholipase A_2_ receptor showed a relatively lower positive rate of only 75.0% in the FSGS+ group in comparison with the positive rate of 90.3% in the FSGS- group (p=0.031). The prognosis was generally similar between the two groups. Among patients who were given non-immunosuppression treatment, those with focal segmental glomerulosclerosis lesions took a relatively longer period of time to achieve complete remission (29.3±7.0 m vs. 15.4±8.9 m, p=0.025) and experienced a higher rate of renal function deterioration (37.5 vs. 5.4%, p=0.033) compared with the other ones. While among those receiving immunosuppression treatment, both groups received similar remission rates.

**CONCLUSION:**

Compared with FSGS- group, idiopathic membranous nephropathy with focal segmental glomerulosclerosis lesions represented more severe nephrotic syndrome and worse renal function. In view of the renal function decline during the follow-up, more aggressive treatment with the use of immunosuppressants should be considered for idiopathic membranous nephropathy patients with focal segmental glomerulosclerosis lesions.

## INTRODUCTION

Idiopathic membranous nephropathy (IMN), which is one of the leading causes of nephrotic syndrome in adults, continued to update during the past two decades^1^, especially after M-type phospholipase A_2_ receptor (PLA_2_R) was identified as a major target antigen in 70% of IMN^2^. Among patients who have been diagnosed with IMN, different situations may occur: spontaneous remission, persistent nephrotic syndrome with preserved renal function, or refractory proteinuria with worsening renal function. Some may even develop end-stage renal disease (ESRD). Various features have been shown to predict unfavorable course in IMN patients, including male sex, heavy proteinuria, renal insufficiency at presentation, hypertension, age, and degree of interstitial fibrosis^3-7^. The prognostic indicators of IMN are still needed to be studied to predict the outcomes, help choose individual therapy, and weigh the advantages and disadvantages of different therapies. With the in-depth study of the pathogenesis and clinical features of membranous nephropathy, the correlation between pathological features and prognosis has been found gradually.

Since the first report of IMN with focal segmental glomerulosclerosis (FSGS) lesions pathologically by Churg and Ehrenreich in 1973^8^, the clinical and pathological features of these patients have received increasing attention. The incidence rate is 10–43% according to the current reports^9-16^. A few studies have stressed the poor prognostic meaning of FSGS lesions with IMN^10,11,13,16,17^. According to the previous reports, patients with FSGS lesions showed higher serum creatinine levels and more severe nephrotic syndrome. While some other research and meta-analysis did not support this conclusion^3,14,18^, some research demonstrated the relationship between the specific FSGS lesions and the renal outcome, such as non-atypical lesions (pure synechia, segmental hyperplasia of podocytes or thickening of the GBM accompanied by proliferation of the mesangial matrix, and absence of typical FSGS) and non-glomerular tip lesion^19,20^. In addition, the previous studies were less focused on the treatment efficiency of those with FSGS lesions. Because of the heterogeneity of the study design and the small sample size among those studies, there was still no uniform conclusion, and more research is still required. In this study, we aimed to ascertain the clinical and pathological characteristics of IMN with and without FSGS lesions and analyze the outcomes and treatment efficiency of the two groups.

## METHODS

### Study design

This study was designed as a retrospective cohort study. We used the data who were diagnosed as IMN at Huashan Hospital affiliated with Fudan University and Wuxi People's Hospital. The data underlying this study were collected from the medical record system of the two centers. The study received local ethics committee approval (approval number and date: KY2016-394, February 6, 2017), and all patients gave written informed consent.

### Patients selection

Briefly, patients who were diagnosed as IMN pathologically by renal biopsy between January 2008 and December 2014 with ages above 18 years and gender unlimited were enrolled. Exclusion criteria included secondary MN, such as V-type lupus nephritis, hepatitis B, hepatitis C, malignancy, syphilis, autoimmune conditions such as Sjogren syndrome, rheumatoid arthritis, ankylosing spondylitis, and anti-glomerular basement membrane disease. Patients without FSGS lesions, if the number of glomeruli obtained by renal biopsy was less than 8, were also excluded to avoid bias due to missed diagnosis. Patients who were biopsy-proven superimposed FSGS lesions were selected as a study group. Those without FSGS lesions were selected for comparison.

### Clinical and laboratory data

Data such as gender, age, medical history, medications, serum creatinine, serum albumin, cholesterol, 24-h urinary protein excretion, and systolic and diastolic blood pressure were collected retrospectively according to the medical records at the time of biopsy. The estimated glomerular filtration rate (eGFR) was calculated with the CKD-EPI creatinine equation. In patients who were followed up at the two centers, treatment was divided into two classes: (1) non-immunosuppressive therapy with symptomatic approaches included angiotensin-converting enzyme inhibitors (ACEIs) or angiotensin receptor blockers (ARBs) and (2) immunosuppressive therapy included glucocorticoid combined with immunosuppressant (cyclophosphamide or calcineurin inhibitors). Serum creatinine, serum albumin, and 24-h urinary protein excretion were measured at least every 6 months.

### Pathological evaluation

All biopsy specimens were processed with light microscopy, immunohistochemistry, and electron microscopy using standardized techniques. The biopsies were evaluated in detail for the following features: total number of glomeruli, crescents, global sclerosis, FSGS lesion, the extent of interstitial fibrosis, and the degree of arteriosclerosis. Focal glomerulosclerosis was defined as a focal lesion with mesangial matrix expansion leading to the collapse of the glomerular capillary loops. Criteria of Columbia classification of FSGS were used for classification.

### Renal PLA_2_R staining

Renal tissues from 163 of the patients were stained for the PLA_2_R antigen. PLA_2_R was detected in 2-μm frozen sections using rabbit polyclonal anti-PLA_2_R antibodies (Sigma, America, 295631) at a dilution of 1:500 followed by donkey anti-rabbit IgG (Millipore, America, AP182F) at a dilution of 1:100. PLA_2_R staining was considered positive if there was positive granular staining along the capillary loops of glomeruli, and negative if there was no staining in the glomeruli.

### Outcomes

Follow-up was started at the time of biopsy and continued until July 2016. We analyzed the remission, relapse, and renal function of those who were followed up for over 12 months. CR was defined by proteinuria <0.3 g/day, with normal serum albumin level and renal function. Partial remission (PR) was defined by proteinuria <3.5 g/day or descending over half the peak level with normal renal function. Otherwise, no remission (NR) was diagnosed. Relapse of proteinuria was defined as recurrent proteinuria within the nephrotic range or over half the peak level. Renal function deterioration was defined as the last eGFR descending over 30% compared with eGFR at baseline.

### Statistical analysis

Statistical analysis was performed with SPSS version 13.0 for Mac. For continuous variables, normal distribution was examined by the Kolmogorov-Smirnov (K-S) test. T-test and Mann-Whitney U test were applied for normal distribution variables and non-normal distribution variables, respectively. For categorical variables, correlations were calculated using the chi-square test and Fisher exact test, if appropriate. For all analyses, p<0.05 was considered significant.

## RESULTS

### Clinical and laboratory characteristics

Finally, 236 patients were included. A total of 60 cases were accompanied by FSGS lesions (FSGS+ group), and 176 cases were not (FSGS- group). Table 1 shows the baseline characteristics of the two study groups. There is no difference in gender distribution and mean age between the two groups. FSGS+ group shows a much higher percentage of the history of hypertension and a higher median systolic blood pressure level at the time of renal biopsy. The two groups were similar with respect to diastolic blood pressure. Patients in the FSGS+ group represented more severe nephrotic syndrome with a higher 24-h proteinuria of a median level of 5.61 g/24 h and a lower serum albumin of a median level of 20.4 g/L than the FSGS- group. Also, the cholesterol level showed the same trend. Meanwhile, there was a significant decline in serum creatinine and eGFR levels in FSGS+ cases compared with FSGS- ones.

**Table 1:** Comparison of the medical history, laboratory parameters, and pathological characteristics between the FSGS+ and FSGS- groups.

		FSGS+ (n=60)	FSGS- (n=176)	p
Gender (M%)		65.0%	56.3%	NS
Age (years)		52.4±16.3	51.8±15.1	NS
History of DM (%)		15.3%	17.0%	NS
History of HBP (%)		38.3%	20.0%	0.004
Systolic BP (mmHg)		137 (120, 160)	130 (120, 140)	0.009
Diastolic BP (mmHg)		80 (78, 90)	80 (75, 90)	NS
Albumin (g/L)		20.4±7.8	24.5±6.7	<0.001
24-h proteinuria (g/day)		5.61 (3.10, 7.87)	3.82 (2.31, 5.79)	0.002
BUN (mmol/L)		4.5 (3.6, 6.0)	4.3 (3.5, 5.7)	NS
Serum creatinine (μmol/L)		80.8 (65.8, 97.9)	72.0 (58.7, 84.9)	0.003
eGFR (mL/min/1.73 m^2^)		86 (66, 101)	95 (81, 108)	0.007
UA (mmol/L)		0.345±0.086	0.364±0.092	NS
Glomeruli (n)		15.5 (12.0, 22.0)	18.5 (13.0, 25.8)	NS
Obsolescent glomeruli (%)		6.5% (4.5%, 8.3%)	5.4% (3.9%, 7.7%)	NS
Crescent (%)		13.3%	2.8%	0.005
Tubular atrophy	0	17 (28.3%)	61 (34.7%)	
	1	28 (46.7%)	89 (50.6%)	
	2	13 (21.7%)	24 (13.6%)	
	3	2 (3.3%)	2 (1.1%)	NS
Interstitial fibrosis	0	14 (23.3%)	59 (33.5%)	
	1	28 (46.7%)	89 (50.6%)	
	2–3	18 (30.0%)	28 (15.9%)	0.045
Cast	0	5 (8.3%)	43 (24.4%)	
	1	37 (61.7%)	87 (49.4%)	
	2	18 (30.0%)	46 (26.1%)	0.022
MN stage	Early stage and stage I	19 (31.7%)	85 (48.3%)	
	Stage I–II	10 (16.7%)	43 (24.4%)	
	Stage II	18 (30.0%)	42 (23.9%)	
	Stage II–III	8 (13.3%)	3 (1.7%)	
	≥Stage III	1 (1.7%)	1 (0.5%)	0.001
Renal PLA_2_R +(%)		45 (75.0%)	159 (90.3%)	0.031

Values are n (%), mean (SD), and median (interquartile ranges). M: male; DM: diabetes mellitus; HBP: high blood pressure; BP: blood pressure; BUN: blood urea nitrogen; eGFR: estimated glomerular filtration rate; UA: uric acid; CHO: cholesterol; PLA_2_R: phospholipase A_2_ receptor; NS: no significant.

### Pathological characteristics

With respect to pathological findings, there was no difference in the number of glomeruli between the two groups. Patients in the FSGS+ group presented a higher frequency of crescents, higher interstitial fibrosis and tubular level, and a higher proportion of obsolescent glomeruli, although the latter was not statistically significant. In the FSGS- group, the most common stage of MN was early stage I (48.3%). In comparison, the FSGS group showed a higher level of stage II to stage III (Table 1). According to Columbia's classification of FSGS, the most common cases were classified as no otherwise specified (NOS) seen in 66.67% of the FSGS+ group. Notably, 28.33% were Tip and 5.00% were perihilar (PH). No collapsing or cellular lesion was noted.

### Prognostic characteristics

A total of 25 patients and 104 patients were followed, respectively. No difference was observed between the two groups neither in the remission rate nor in the relapse rate. Notably, 6 patients in the FSGS+ group (24.0%) and 10 in the FSGS- group (9.6%) had their eGFR descending over 30% during follow-up (p=0.050). eGFR at the latest follow-up was also lower in the FSGS+ group, although there was no statistical difference (Table 2).

**Table 2:** Comparison of prognostic characteristics between the FSGS+ and FSGS- groups.

	FSGS+ (n=25)	FSGS- (n=104)	p
Period of follow-up (months)	25.0 (20.0, 39.5)	24.0 (19.0, 36.0)	NS
Outcome-NR	4 (16.0%)	27 (26.0%)	
Outcome-PR	10 (40.0%)	35 (33.7%)	
Outcome-CR	11 (44.0%)	42 (40.3%)	NS
Time between biopsy and PR (months)	7.4 (5.4)	10.2 (6.6)	NS
Time between biopsy and CR (months)	16.6 (9.8)	12.3 (6.4)	NS
Relapse (%)	6 (28.6%)	14 (24.7%)	NS
eGFR at the latest follow-up	75.9 (33.3)	85.9 (26.4)	NS
eGFR descending>30%	6 (24.0%)	10 (9.6%)	0.050

Values are n (%), mean (SD), and median (interquartile ranges). PR: partial remission; CR: complete remission; eGFR: estimated glomerular filtration rate; NS: no significant.

### Prognosis situations with different treatments

A total of 8 patients in the FSGS+ group and 37 patients in the FSGS- group received non-immunosuppressive therapy. These patients presented a relatively lower level of 24-h urinary protein of mean 4.6±4.3 and 3.3±2.5 g/day. Both groups showed more than 60% of spontaneous remission. However, in the FSGS+ group, an additional 13.3 months on average was needed to achieve CR, and an obvious decline in eGFR was presented at the latest follow-up. Two patients in the FSGS+ group even progressed to ESRD. In comparison, patients with no combined FSGS lesion presented a relatively lower eGFR descending rate and a well-preserved renal function, which was similar to the eGFR level at baseline. This result indicated that renal function deterioration was more likely to occur in IMN patients combined with FSGS lesions if non-immunosuppression therapy was accepted; also, it would take much longer for them to achieve PR or CR. For those who achieved immunosuppressive therapy, no difference was observed in remission or renal function (Table 3).

**Table 3:** Prognostic characteristics between the FSGS+ and FSGS- groups with non-immunosuppressive and immunosuppressive therapy.

		Non-immunosuppressive therapy			Immunosuppressive therapy		
		FFSGS+ (n=8)	FSGS- (n=37)	p	FSGS+ (n=17)	FSGS- (n=67)	p
Period of follow-up (months)		39.6±25.3	30.1±16.1	NS	23.0 (19.5, 33.0)	24.0 (18.0, 36.0)	NS
Outcome (%)	NR	3 (37.5%)	13 (35.1%)		1 (5.9%)	14 (20.9%)	
	PR	12 (25.0%)	11 (29.7%)		8 (47.1%)	24 (35.8%)	
	CR	3 (37.5%)	13 (35.1%)	NS	8 (47.1%)	29 (43.2%)	NS
Time from biopsy to PR (months)		11.3±5.0	15.5±7.7	NS	6.4±5.2	8.1±4.8	NS
Time from biopsy to CR (months)		29.3±7.0	15.4±8.9	0.025	11.8±5.2	11.0±4.5	NS
Relapse (%)		0 (0.0%)	1 (4.2%)	–	6 (37.5%)	18 (34.0%)	NS
eGFR at the latest follow-up		66.3±45.0	91.2±28.8	NS	81.9±27.4	83.0±24.7	NS
eGFR descending>30%		3 (37.5%)	2 (5.4%)	0.033	3 (17.6%)	8 (11.9%)	NS

Values are n (%), mean (SD), and median (interquartile ranges). Alb: albumin; NR: no remission; PR: partial remission; CR: complete remission; NS: no significant.

## DISCUSSION

Patients with IMN would greatly benefit if some clinical or pathological characteristics could predict disease prognosis with high accuracy. It is still insufficient for making a decision on what therapy should be chosen and when to start immunosuppressive therapy.

Idiopathic membranous nephropathy with FSGS lesions was first reported by Churg and Ehrenreich^8^. Dumoulin et al., mentioned in his study that FSGS lesions on IMN portended a significantly worse outcome in terms of nephrotic syndrome and renal insufficiency^16^. A recent study published in 2014 also concluded that FSGS lesions predict renal outcomes independently of clinical data in nephrotic IMN patients with decreased renal function^17^. However, there has been no consensus yet. According to another two studies^3,14^, there is no significant difference between patients with or without FSGS lesions on remission, renal insufficiency, and ESRD. FSGS is not an accurate prognostic marker in IMN.

We reviewed the medical history of 236 IMN patients in two medical centers and found that patients with FSGS lesions presented heavier nephrotic syndrome and relatively lower eGFR. Pathologically, the FSGS+ group presented a higher frequency of crescents and a greater degree of interstitial fibrosis and tubular atrophy. The eGFR level declined more severely in the FSGS group than the other, which indicates that IMN combined with FSGS lesion may portend poorer renal outcome.

In our study, we observed a relatively higher remission rate with a relatively lower dose of proteinuria. According to KDIGO clinical practice guideline for glomerulonephritis in 2012, on the basis of antihypertensive and antiproteinuric therapy during an observation period of 6 months, for patients with nephrotic syndrome and urinary protein excretion persistently NR, initial immunosuppressive therapy may be started. However, our study points out that for IMN patients with proteinuria levels of 3–5 g/L, the observation period could be extended to 1 year and an additional 30% remission might be observed. The FSGS+ group took an average of 29.3 months to achieve CR, which meant those patients might have had more exposure to a series of potential complications caused by nephrotic syndrome. At the latest follow-up, renal function decreased significantly in FSGS+ IMN. It inferred that the poor renal outcome may be correlated to the delayed remission of proteinuria, which may be inclined to more aggressive therapy for patients superimposed with FSGS lesions to achieve remission and protect renal function. However, physicians must have to weigh the pros and cons of different therapies to make a rational decision.

Phospholipase A_2_ receptor was characterized as a major target antigen of idiopathic MN. It may be a biomarker for identifying whether it is idiopathic or not. Serum anti-PLA_2_R antibodies (PLA_2_R-Ab) are detected in a majority of patients with IMN, and the antibody titer is associated with disease activity and prognosis. Those who had low levels of anti-PLA_2_R antibodies were prone to developing remission within a shorter period^21-24^. There are few studies on PLA_2_R and FSGS combined IMN. According to our study, both groups had a high positive rate. While 75.0% of FSGS+ patients showed PLA_2_R positive, which was significantly lower compared with the FSGS- group. The result may indicate that PLA_2_R perhaps may not completely explain the pathogenesis of the FSGS lesion.

Our study has several limitations. First, as it is a retrospective study, some patients were lost during the follow-up, which may cause a selective bias. The follow-up sample size of the FSGS+ group receiving non-immunosuppressive therapy was too small to draw stronger conclusions, so our analyses should be treated with caution, and further work with larger sample sizes is warranted. Second, in our study, we describe a more severe disease status of IMN with FSGS lesions, yet we did not have enough samples to further analyze the relationship between prognosis and different types of FSGS lesions, and the mechanism underlying the FSGS lesion remains unclear. More research is still needed.

## CONCLUSION

Idiopathic membranous nephropathy with FSGS lesions presents more severe nephrotic syndrome and worse renal function at baseline. Renal function may decline faster, especially for those who receive non-immunosuppressive therapy, which may indicate that more aggressive treatment to use immunosuppressants to achieve proteinuria remission and protect renal function should be considered for IMN patients with FSGS lesions.
